# High incidence of vitamin D deficiency in 2 – 17 year olds presenting with fracture to a Melbourne suburban public hospital

**DOI:** 10.1186/1687-9856-2015-S1-P59

**Published:** 2015-04-28

**Authors:** Dae Kwon, Chris Harris, Abhay Khot, David Krieser, Danny Liew, Sharon Brennan, Peter Ebeling, Christine Rodda

**Affiliations:** 1 NorthWest Academic Centre, The University of Melbourne, Sunshine Hospital, St Albans, VIC, Australia; 2 Paediatric Orthopaedic Unit Sunshine Hospital, St Albans, VIC, Australia; 3 Paediatric Emergency, Paediatric Unit, Sunshine Hospital, St Albans, VIC, Australia; 4 The Melbourne Epi Centre, University of Melbourne, Royal Melbourne Hospital, Parkville, VIC, Australia; 5 Epidemiology Unit for Musculoskeletal and Metabolic Disorders (Epi-UMMD) Faculty of Health, Deakin University, Geelong, VIC, Australia; 6 Department of Medicine Monash University, Monash Medical Centre, Clayton, VIC, Australia

## 

To determine vitamin D deficiency risk and other lifestyle factors in children and teenagers aged 2 – 17 years presenting with fracture to Sunshine Hospital, a clinical observational study was undertaken using convenience sample data collected from children and teenagers aged 2 - 17 years of age presenting with fracture, for whom consent had been obtained to determine clinical characteristics and lifestyle factors. Recruitment was undertaken over a 4 month period 1^st^ February to 31st May 2014. A suburban Melbourne (latitude 38^0^S) teaching hospital, Sunshine Hospital provides paediatric orthopaedic services for a high proportion of children and teenagers from ethnically diverse backgrounds with an increased proportion of highly pigmented individuals, which may influence vitamin D status specifically. Proxy measures of vitamin D were used (skin pigmentation, hours spent outdoors, sunscreen use and obesity) [1] to determine patients at risk for Vitamin D deficiency. Further consent was then obtained from at risk patients to take blood for 25 OH vitamin D (LIAISON^®^, Diasorin Assay). Of the 162 patients recruited into this study, 133 (82%) had risk factors for vitamin D deficiency. Of these 108 (81% of at risk) consented to blood testing for 25 OH vitamin D, with a median of 50nmol/l (range 14 – 110nmol/l) obtained. A total of 56 (52% at risk, 34% of total participants) were found to be vitamin D deficient and of these 45 (80% at risk) were mildly deficient (25 OH D 30 -50nmol/l) and 11 (20% at risk) had moderate deficiency (25 OH D 12.5 – 29 nmol/l). Although our study was undertaken at the end of summer, one third of the patients in our study were vitamin D deficient. Furthermore, half of those clinically deemed at risk for vitamin D deficiency were confirmed on biochemical testing. Childhood fracture incidence has been reported to be increasing, and with relatively stable genetic characteristics, any variations in childhood fracture would imply environmental changes. The effect of mild to moderate vitamin D deficiency on fracture risk, healing and longer term refracture risk in children and teenagers is yet to be determined, however, based on our findings we recommend that vitamin D status be assessed in all at risk children and teenagers living in urban environments at higher latitudes presenting with fracture.
